# Power Quality Disturbance Tracking Based on a Proprietary FPGA Sensor with GPS Synchronization

**DOI:** 10.3390/s21113910

**Published:** 2021-06-05

**Authors:** Oscar N. Pardo-Zamora, Rene de J. Romero-Troncoso, Jesus R. Millan-Almaraz, Daniel Morinigo-Sotelo, Roque A. Osornio-Rios, Jose A. Antonino-Daviu

**Affiliations:** 1 Faculty of Engineering, Autonomous University of Queretaro, San Juan del Rio 76806, Mexico; opardo10@alumnos.uaq.mx (O.N.P.-Z.); troncoso@hspdigital.org (R.d.J.R.-T.); raosornio@hspdigital.org (R.A.O.-R.); 2 Faculty of Physical, and Mathematical Sciences, Autonomous University of Sinaloa, Culiacan 80040, Mexico; jrmillan@uas.edu.mx; 3 Department of Electric Engineering, University of Valladolid, 47011 Valladolid, Spain; daniel.morinigo@eii.uva.es; 4 Instituto Tecnológico de la Energía, Universitat Politecnica de Valencia, 46022 Valencia, Spain

**Keywords:** global positioning system, industrial facilities, propagation, power quality disturbance, particle swarm optimization, genetic algorithms, field-programmable gate array

## Abstract

The study of power quality (PQ) has gained relevance over the years due to the increase in non-linear loads connected to the grid. Therefore, it is important to study the propagation of power quality disturbances (PQDs) to determine the propagation points in the grid, and their source of generation. Some papers in the state of the art perform the analysis of punctual measurements of a limited number of PQDs, some of them using high-cost commercial equipment. The proposed method is based upon a developed proprietary system, composed of a data logger FPGA with GPS, that allows the performance of synchronized measurements merged with the full parameterized PQD model, allowing the detection and tracking of disturbances propagating through the grid using wavelet transform (WT), fast Fourier transform (FFT), Hilbert–Huang transform (HHT), genetic algorithms (GAs), and particle swarm optimization (PSO). Measurements have been performed in an industrial installation, detecting the propagation of three PQDs: impulsive transients propagated at two locations in the grid, voltage fluctuation, and harmonic content propagated to all the locations. The results obtained show that the low-cost system and the developed methodology allow the detection of several PQDs, and track their propagation within a grid with 100% accuracy.

## 1. Introduction

Nowadays, power quality (PQ) is a combination of characteristics and conditions of the power supplied to the equipment to guarantee its continuous operation, and its studies are important for industrial processes to maintain the quality standards of the power grid and to avoid damage to equipment connected to the grid [1]. Disturbances cause a poor PQ and are produced by non-linear loads connected to the grid; these sources can be connected at long distances from the point of interest to be studied. The propagation of disturbances strongly depends on the topology of each grid and the impedance, so disturbances can decay with distance from their point of origin, but they can also be amplified [2]. There are commercial devices that can measure and analyze electrical signals; for example, the SEL-735 PQ and Revenue Meter [3] is a modular PQ meter which allows the capture of power quality disturbances (PQDs), but does not allow synchronized measurements to monitor the propagation of disturbances, and it is expensive. The Fluke 1760TR PQ Analyzer [4] is a single point measurement device that can synchronize measurements with other devices, but it can only measure certain PQ parameters for short periods of time, so it cannot perform continuous PQ monitoring, nor is it suitable for monitoring the propagation of disturbances because it requires the acquisition of several devices, and it is a high-cost device. Due to the mentioned limitations of commercial equipment, it is important to develop a system that detects all PQDs and performs synchronized measurements to monitor the propagation of disturbances, and is cost-effective.

Aiming to analyze PQDs, different signal processing techniques have been developed over the years to perform the detection, classification, and quantification of PQDs using different mathematical models developed for each one depending on its application. The authors in [5] performed a compilation of detection and classification techniques for PQDs generated by renewable energy sources in a grid. This work provides knowledge of the latest techniques developed for PQ diagnosis. The authors in [6] provided a comprehensive review on the state-of-art techniques based on digital signal processing and machine learning for the automatic recognition of PQ events. The authors in [7] developed a structured methodology in combination with a mathematical model which describes waveforms that contain simultaneous PQDs capable of being adjusted to reproduce PQDs contained in electric waveforms. This model was tested with recorded signals, and proved to be able to reproduce the signals with minimal error. Nevertheless, the extraction process is semi-automatic, and requires the support of other techniques for this purpose. The authors in [8] introduced a wavelet-based PQ indicator. An instantaneous disturbance index (ITD) and global disturbance ratio index (GDR) are defined to integrally reflect the PQ level in the power distribution network under steady-state and/or transient conditions. The effectiveness of this method has been proved by comparing the proposed PQ indicators with classical indices, and the results confirm that the method efficiently extracts the characteristics of each component from the multi-event test signal, but it has not been implemented in hardware. In [9], the authors submitted a method of detection and classification of PQDs using different wavelets and neural network classifiers. Different wavelets were used to extract features of the raw signals, and a neural network was used to detect the PQDs. Simulation results showed the performance of the network for different wavelets and its efficacy, but improved accuracy can be obtained if the Fuzzy Technique is employed for the detection of PQDs. Some detection and classifications techniques have been implemented in PQ measurement systems to detect fault events in real time, whereas the authors in [10] proposed a smart sensor that allows the detection, classification, and quantification of PQDs, where it uses Hilbert transform techniques for detection, a feed forward neuronal network (FFNN) for classification, and a real mean square (rms) voltage, peak voltage, crest factor, and total harmonic distortion (THD) to quantify the disturbances. The techniques have been integrated into a methodology that allows the online processing of a single PQD, and this has been validated and tested with synthesized signals and under real operating conditions.

There are research papers that report propagation studies of specific disturbances; in [11], the authors studied through simulations the propagation of only flicker phenomena in a system supplied by a wind farm, and it was observed that, even if the wind turbines are considered synchronized and producing the maximum level of voltage fluctuation, the flicker indexes are below the limits imposed by current standards. The authors in [12] realized a simulation to study the PQ and stability during a fault event propagated in a microgrid using MATLAB. The outcome was that the placement of the fault and the type greatly affect the stability of an autonomous microgrid. The fault propagation study was performed only in simulation using MATLAB, so it has not been implemented in hardware. In [13], the authors developed an efficient compressive sensing harmonics detector (CSHD) to identify and estimate the principal pollution source of harmonics, which is simulated and validated by means of appropriate testing to be performed on an example IEEE 13 bus distribution grid, thus obtaining an efficient and accurate CSHD for the identification and estimation of the main harmonic sources in a grid. In [14], the authors analyzed the impact of a distributed generation unit on a power grid, and then energy storage systems (ESS) of different capacities were integrated into the power grid in an effort to study the improvements in the PQ. The obtained results showed that the integration of energy storage systems into the power grid improves the PQ of the grid, which can be extremely useful for system operators. In [15], the authors realized an economic study to analyze the economic feasibility for the integration of flywheel ESS in a wind power plant. The integration of ESS in the wind farm will allow an increase in the load factor of the power plant by cutting down the probability of being disconnected from the power grid for impacting the stability of the network. However, the integration is only feasible with the government subsidy in renewable energy projects. The above-mentioned works perform propagation and source detection studies of PQDs in simulations, so it is necessary to implement some disturbance detection techniques in hardware. The authors in [16] used tens of thousands of balancing electricity meters for measuring the quality of the electricity supply indicator and identifying the source of PQDs based on the analysis of 10 min added data from the distributed measurement system. This article shows that the system of the balance meter equipped with the PQ functionalities can provide a wide spectrum of grid monitoring and diagnosis capabilities. However, this study is not always suitable for other cases as it uses high-cost commercial equipment. The authors in [17] presented a feasibility study on the procedure to implement PQ metrics in a low-cost smart metering platform by the use of commercial modules for PQ measurement, and performed a harmonic analysis. The results were delivered by choosing a real case study of STCOMET, a commercial electronic board widely used for remote energy metering purposes, and the paper has verified the possibility to implement PQ metrics in it. The authors of [18] designed an intelligent street lighting PQ monitoring system to test the adaptive current control strategy, in which a commercial measurement system from National Instruments (NI) cDAQ-9185 was used. As a result, they observed that the THD decreased by 19% on average. The above-mentioned works aimed to identify the main sources of a specific disturbance in each case by the use, in some occasions, of high-cost commercial equipment. However, it is important to monitor all disturbances and study how they propagate in the grid, since this is essential to obtain an accurate synchronization between the devices that allows the monitoring of different points of the grid at the same time.

There are initiatives to monitor disturbances in the grid, but they present some disadvantages. This notion PV-on time represents a local definition, in [19], and in fact is about real-time monitoring of the plant, which has been developed to supervise the operation mode of a Grid-Connected Utility-Scale photovoltaic power plant in order to ensure the reliability and continuity of its supply. This system utilizes sensors distributed in the plant to obtain a PQ analysis in real-time; the data acquisition equipment has been integrated with a precision time protocol (PTP) to synchronize the collected data with a nanosecond level of synchronization, yet it has the disadvantage that the integration turns out to be an expensive wired infrastructure. The authors in [20] developed an open architecture smart sensor network, based on field-programmable gate array (FPGA) technology, which is capable of monitoring PQ continuously in industrial facilities, public buildings, and residential buildings. It is also capable of estimating different PQ indices, as well as identifying disturbances and detecting connection and disconnection events, and it is also capable of locating events in a synchronized way in different points of an electrical installation by using a real-time clock (RTC) that allows a synchronization of the measurements in different points of the grid. Nevertheless, this synchronization has problems because the RTC of each device has different oscillators which, during long time measurements, cause a desynchronization. The authors of [21] developed a measurement technique to detect rapid voltage changes (RCV) and their propagation effect in an electrical distribution grid; this technique has the ability to detect RCV disturbances and correlate them with other electromagnetic interference events simultaneously, where four PQ analyzers, model PQube 3 manufactured by PSL—Power Standards Lab, were used for data acquisition while Raspberry Pi-3 devices were used to obtain the time reference, obtaining as a result the probability that an RCV in an electrical distribution grid generates a sag in an uninterrupted source—70% to 93.8%. Using the Raspberry Pi as a time reference involves using the ethernet or WIFI port to keep the Raspberry Pi clock synchronized, which means additional cost to the grid infrastructure. The authors in [22] used a phasor measurement unit (PMU) for harmonic state estimation for an unbalanced three-phase distribution system. A PMU utilizes a global positioning system (GPS) to perform synchronized measurements. This allows the identification of harmonic sources and the monitoring of harmonic components, and their propagation in the grid. PMUs are devices that estimate the magnitude and phase angle of the voltage and current signals in the power grid, and are high-cost devices. Owing to the limitations of the devices, it is important to develop a system capable of synchronizing measurements that can accurately detect and classify PQDs, and that can detect whether a disturbance has propagated throughout the network.

The aim of this work is to develop a system to detect whether a disturbance is generated at a point in the grid, and to detect to which point it has propagated within the grid in the industrial facility; a proprietary system based on an FPGA sensor has been developed to perform synchronized measurements with other proprietary systems using a GPS to track the propagation of PQDs. The proposed method uses a parameterized model based on genetic algorithms (GAs) and particle swarm optimization (PSO), which describes the components of all PQDs to decompose the current or voltage signal to detect disturbances. GPS allows the synchronization of the measurements acquired by the data logger, called GSD in the experimental setup. This synchronization allows the tracking of the propagation of disturbances within the grid. This method has been validated in an industrial facility, installing three systems at different points of the network to detect disturbances propagating through it, and an analysis of the propagated disturbances (transients, voltage fluctuation, and harmonic content) has been carried out.

The contribution of this work is summarized in a punctual form as shown below.

- This paper describes a low-cost proprietary system which implements a methodology to track the propagation of PQDs by performing synchronized measurements, using GPS, between different proprietary data loggers located at different points in the grid. This is due to the fact that other systems perform punctual PQ analysis of a certain number of PQDs, and some other works do not use GPS, using other synchronization techniques which have limitations that compromise the synchronization of the measurements.
- This paper shows the hardware implementation of a full PQD parameterized model based on PSO and GAs that allows the detection and classification of several PQDs. Other works only allow the detection of a limited number of PQDs and perform offline detection.

The contents of this paper are structured in different sections: Section 2 presents the theoretical background, and some concepts that are an important part of the theory within; Section 3 presents the hardware used in the paper; Section 4 presents the development of the methodology for the detection and tracking of disturbances; Section 5 shows the results obtained from the measurements performed in the industrial facility; Section 6 discusses the results obtained; Section 7 presents the conclusions of this paper.

## 2. Theoretical Background

### 2.1. PQD

PQ is a concept which refers to several parameters such as voltage, current, and frequency that are defined in a range. Any alteration of these parameters is known as a PQD [6]. There are different types of disturbances, such as voltage sag, voltage swell, transient, flicker, harmonics, interruption, among others [23].

Each PQD is described by means of a mathematical model. However, the authors in [7] have developed a mathematical model called a “full PQD parameterized model” that describes all disturbances in one equation shown below.

(1) $$\begin{array}{ll} x\left(t\right)= & X_{CD}+A\left[1+\delta \left(t\right)\right]\{\cos\left[2\pi f_{0}\left(t\right)t+\theta_{1}\left(t\right)\right]\\ & +\overset{N}{\underset{h=2}{\sum }}a_{h}\left(t\right)\cos\left[2\pi hf_{0}\left(t\right)t+\theta_{h}\left(t\right)\right]\}\\ & +\overset{k}{\underset{k=1}{\sum }}b_{k}\left(t\right)\cos\left[2\pi f_{k}\left(t\right)t+\varphi_{k}\left(t\right)\right]\\ & +\overset{M}{\underset{m=1}{\sum }}c_{m}\left[u\left(t-\alpha_{m}\right)-u\left(t-\beta_{m}\right)\right]*e\left[\left(-\frac{t-\alpha_{m}}{\tau_{m}}\right)\right]\\ & *\cos\left[2\pi f_{m}t+\psi_{m}\right]+n\left(t,x_{0},\sigma \right)+\mu \left(t\right) \end{array}$$

Equation (1) classifies PQDs into five types of phenomena defined by their nature; phenomena related to the fundamental frequency amplitude and harmonics, phenomena related to the fundamental frequency with changes in frequency and phase, stationary phenomena non-correlated to the fundamental frequency, transient phenomena, and additive random noise, Gaussian and non-Gaussian [7]. The first term of Equation (1), $X_{CD}$, corresponds to the DC component. The second term has a parameter that controls the behavior of the amplitude A[1+δ(t)], in a fundamental component with a frequency $f_{0}\left(t\right)$, amplitude A, and time-variant phase. $\theta_{1}\left(t\right)$. The third term corresponds to correlated harmonic distortion, which includes time-variant amplitude $a_{h}\left(t\right)$ and phase $\theta_{h}\left(t\right)$. The fourth term describes the non-correlated frequency components, which have time-variant amplitudes $b_{k}\left(t\right)$, phase $\varphi_{k}\left(t\right)$, and frequencies $f_{k}\left(t\right)$. The fifth term is related to fast transients, i.e., short-time transients, between twice as many marks $\alpha_{m},\;\mathrm{and}\;\beta_{m}$. These transients include coefficients with amplitude $c_{m}$ and have an exponential or an oscillatory decaying behavior $\tau_{m}$, and the decay has a rate defined by a single frequency $f_{m}$. The sixth term describes Gaussian noise defined in two parameters: $x_{0}$ is the statistical means, and σ is the standard deviation. Finally, the seventh term models non-Gaussian noise as a time-variant function [1].

Equation (1) has the advantage of modeling many types of disturbances, including sags, swells, transients, harmonics, etc. For this work, transient impulsive, voltage fluctuation, and harmonic content disturbances have been found in measurements performed in industrial facilities, and are used to validate the system proposed in this work.

### 2.2. Transient

**Figure 1:**
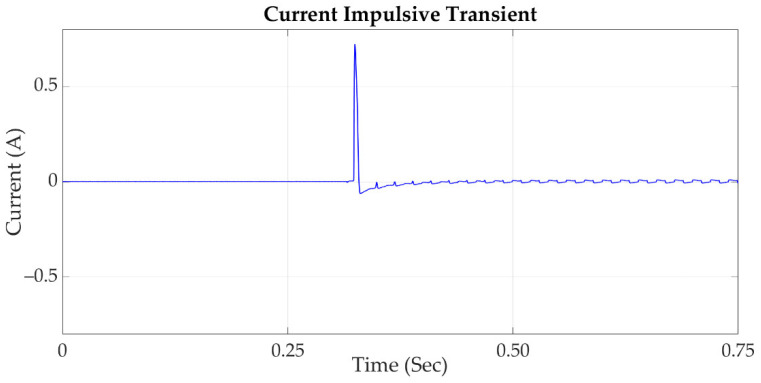
Current signal which contains an impulsive transient.

**Figure 2:**
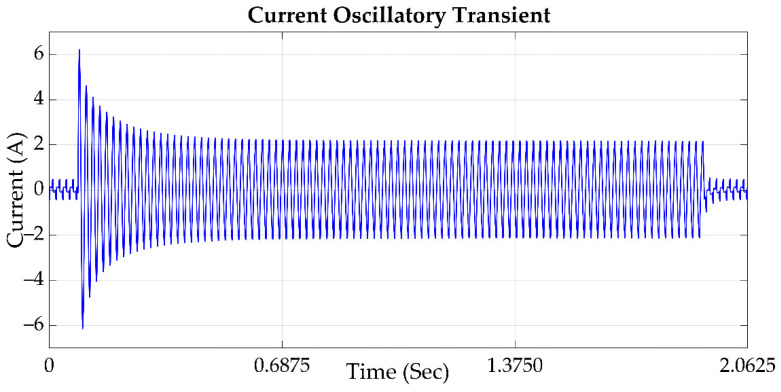
A current signal which contains an oscillatory transient.

According to the IEEE std 1159 definition in [24], a transient is a phenomenon which varies between two consecutive steady states during a short time interval; this disturbance is classified into two categories: impulsive transient and oscillatory transient. In Figure 1, an impulsive transient is shown, being a sudden, non-power frequency change of the nominal voltage or current condition; this disturbance is unidirectional in polarity, and is normally characterized by a short time of rise and a decay. Lightning is the most common cause of impulsive transients. On the other hand, in Figure 2 an oscillatory transient is shown; this is a sudden, nonpower frequency change in the steady-state condition of voltage or current, which includes polarity changes [23]. The transient oscillation occurs in a high-frequency area, and has a characteristic given by a short duration and a wide range of transient frequency domain. Transient faults can lead to the breakdown of a line insulation, power outages, and other serious problems [25].

### 2.3. Voltage Fluctuation

**Figure 3:**
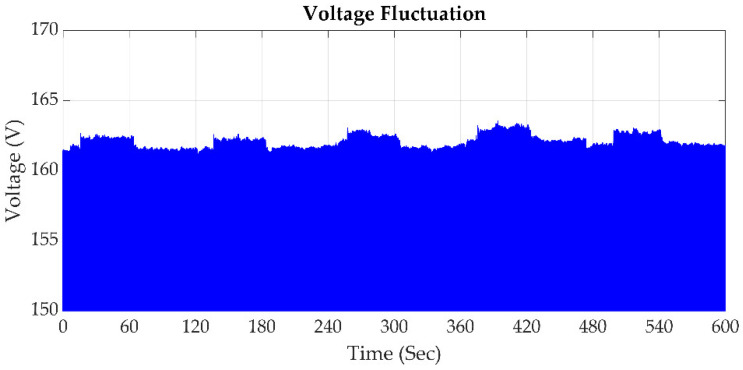
Signal of voltage fluctuation.

According to IEEE std 1159 definition in [24], voltage fluctuation is a series of voltage changes or a cyclical variation in the voltage envelope, as shown in Figure 3, on lamps such that they are perceived to flicker by the human eye, causing irritation and medical problems [26,27]. Sometimes, voltage fluctuations are caused by an arc furnace, motors, rolling mills, mash welders, and electric welders [28].

### 2.4. Harmonic Content

**Figure 4:**
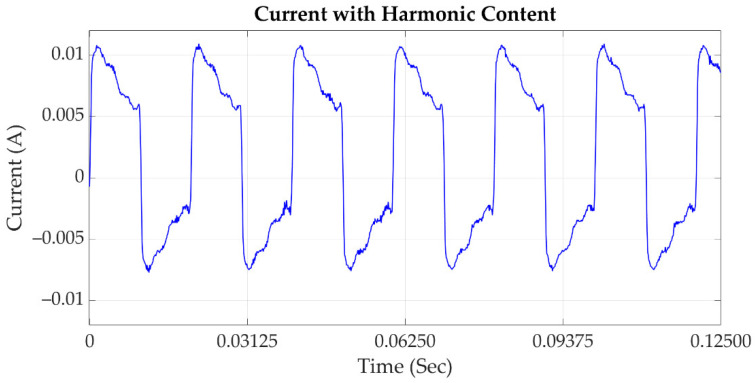
Current signal with harmonic content.

In regard to the IEEE std 1159 definition in [24], harmonic content, shown in Figure 4, relies on sinusoidal voltage or current frequencies which are integer multiples of the fundamental frequency of the grid. The focal sources of harmonics are switching devices, saturable devices, and arcing devices [29]. The presence of harmonics in the grid causes malfunction, and increases reliability problems of the power systems. Sensitive loads connected to the grid are greatly affected by voltage imbalances and harmonics. In the presence of harmonics, motors suffer from overheating, reducing their lifetime, and in transmission lines they cause power imbalance and heating of the lines [30].

### 2.5. Wavelet Transform (WT)

A wavelet is a small wave with oscillatory conditions from which the wavelet functions are generated by the use of a prototype mother wavelet, and a translation τ that corresponds to a window shifting. The WT is depicted in Equation (2), which is a time-scale decomposition technique, also used to obtain the parameter of an input signal at different frequencies; they separate it into their different components [31].

(2) $$\Psi_{m,n}\left(t\right)=\frac{1}{\sqrt{a_{0}^{m}}}\left(\frac{t-nb_{0}a_{0}^{m}}{a_{0}^{m}}\right)$$

where *m* and *n* control the dilation and translation of the wavelet, $a_{0}$ is a dilation step parameter, $b_{0}$ is the location parameter, and $\Psi_{m,n}\left(t\right)$ is the mother wavelet.

### 2.6. Hilbert–Huang Transform (HHT)

HHT is a tool used to track and envelope signals, being able to describe the amplitude changes of a given waveform. Equations (3)–(5) describe this technique.

(3) $$z\left(t\right)=x\left(t\right)+jx_{HT}\left(t\right)=A_{env}\left(t\right)\exp\left(j\theta \left(t\right)\right)$$

(4) $$A_{env}\left(t\right)=\sqrt{x{\left(t\right)}^{2}+x_{HT}{\left(t\right)}^{2}}$$

(5) $$\theta_{env}\left(t\right)=\tan^{-1}\left(\frac{x_{HT}\left(t\right)}{x\left(t\right)}\right)$$

The practical application of the HHT takes place throughout the analytical signal z(t) presented in Equation (3), where $A_{env}\left(t\right)$ is the envelop signal of x(t) as stated in Equation (4), and $\theta_{env}\left(t\right)$ is the instantaneous phase of x(t) as is shown in Equation (5).

### 2.7. Genetic Algorithms (GAs)

GAs are among the most evolved and efficient classes of evolution inspired methods and are based on the key principles of natural evolution theory, and they are also a powerful meta-heuristic technique mainly used in problems where specific values are required, and in which the conditions of the problem are complex. They are also applied where there is lack of data or when previous knowledge on a specific problem is unknown. In such a way, in which parameterized models or functions are impossible to define, another application is the problem. The characteristics of that issue, such as a wide design space, nonlinearity, non-convexity, and multi-objectivity, make it impossible for classical methodologies to find an adequate solution [32].

### 2.8. Particle Swarm Optimization (PSO)

PSO is a technique referred to as a population-based stochastic optimization technique, also used for online and offline monitoring to extract the best subset of features using an extreme learning machine. This technique, in combination with the WT and the HHT, allows finding those parameters of the proposed model, whereas GAs do not.

## 3. Proprietary PQD Detection System

**Figure 5:**
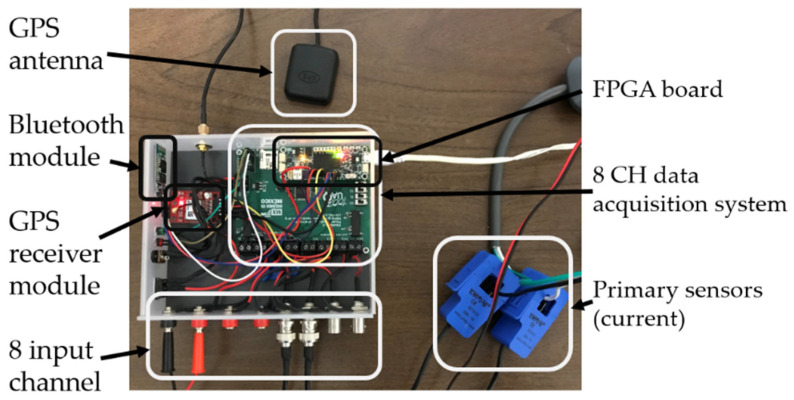
Image of the proprietary GPS (GSD).

**Figure 6:**
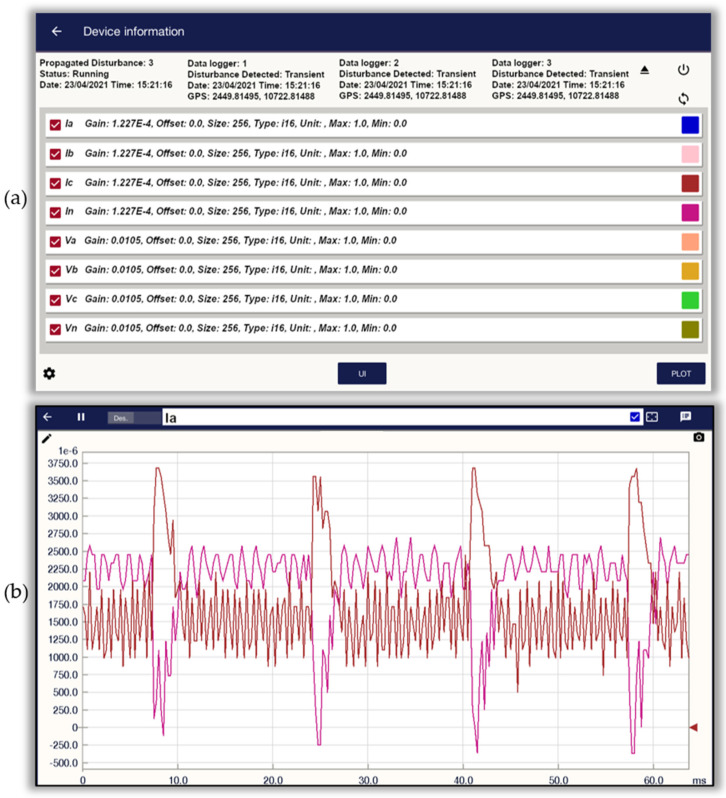
Image interface of the application developed for mobiles to control the proprietary data logger. (**a**) Measurement control screen and configuration of measurement channels: Ia, Ib, Ic, In, Va, Vb, Vc, Vn. (**b**) Real-time signal display screen.

To monitor the propagation of PQDs, it is necessary to develop a system that can detect disturbances and perform synchronized measurements with other devices. The proprietary data logger used is based on Xilinx^®^ FPGA Spartan6 (XC6SLX16, Xilinx, San Jose, CA, USA) with a 16-bit analog-to-digital converter (ADC) with eight channels from Texas Instruments^®^ (ADS130E08) (Texas Instruments, Dallas TX, USA), delivering up to 8000 samples per second, per channel, and synchronizing the measured electrical signals using a pulse-per-second (PPS) provided by a GPS module receiver (U-Blox, Thalwil canton of Zürich, Switzerland). PPS is a broadcast time base synchronized to the satellite atomic clock used to synchronize all data loggers’ internal time base; it obtains a synchronization of the internal time bases of the data loggers. The proprietary data logger with GPS synchronization performs the measurement of four voltage signals (three phases; called Va, Vb, and Vc, and one neutral; called Vn) and four current signals (three phases; called Ia, Ib, and Ic, and one neutral; called In); the signals obtained are stored in a micro-SD memory. It supports a maximum micro-SD memory capacity of 120 GB. The proprietary data logger has a sampling rate of 8000 samples per second and per channel. Figure 5 shows an image of the hardware that constitutes the proprietary data logger developed; the GPS antenna is used to catch the signal from the satellites, the GPS receiver module allows decoding and interpreting the signal received by the GPS antenna, and the Bluetooth module (Qualcomm, San Diego, CA, USA) allows the connection of the data logger with the interface developed for mobiles, as shown in Figure 6, which allows starting and terminating the measurement, as well as seeing in real time the measured voltage and current signals. The data acquisition system allows the digitalization of the eight channels of the input signals, the FPGA board with the algorithms also allows the synchronization of the data logger and the detection of disturbances (also shown), and the primary current sensors allow the detection of the current through a conductor in a non-invasive way, but other current and voltage sensors can be used with a voltage output signal.

The proprietary data logger hardware has been selected based on the purpose and economy of the device—it is important to maintain the cost–benefit ratio without compromising quality. To show the purpose of each specific component used, Table 1 shows the model of each component, its main features, and its functionality for the proprietary data logger.

Figure 6 depicts the screens of the interface developed for mobile devices. This application has been developed in C language, under the linux environment, up to now enabled to be used only for devices with Android operating system. Figure 6a shows the control screen, which is used to initialize and finalize the measurement and, in addition, the initial configuration of gain and offset of each reading channel (Ia, Ib, Ic, In, Va, Vb, Vc, Vn), and the data of the measurement status, date, time, and GPS location are displayed. The developed interface also provides information about the detected disturbance: it indicates the time and which GPS synchronized data logger (GSD) performed the detection. It allows the determination of whether a disturbance has been detected simultaneously in different points within the grid. Figure 6b shows the real time display screen of the signals being measured, allowing the user to select to view one input channel or several input channels simultaneously, distinguishing each signal with a different color.

**Figure 7:**
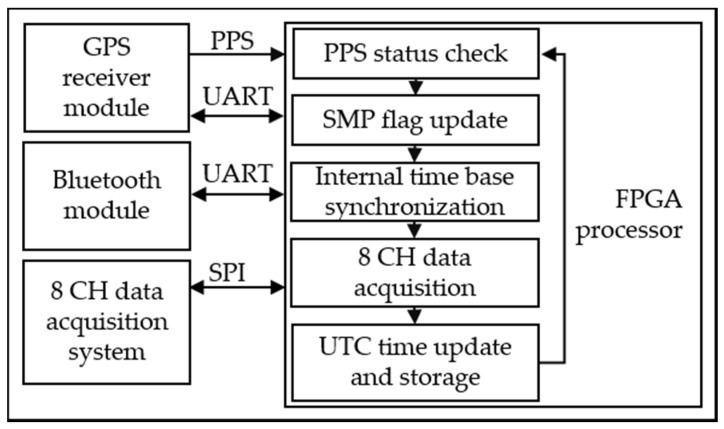
Proprietary data logger with GPS synchronization diagram.

The procedure used to synchronize the measured electrical signals is shown in Figure 7, which describes the procedure of the internal time base synchronization: the first step’s purpose is to verify if the PPS has been received by the GPS receiver module; in the second step, the PPS information is stored in a flag called SMP; in the third stage, the internal time base of the data logger is synchronized using the PPS provided by the GPS receiver module; in the fourth step, the FPGA processor acquires the eight input channels utilizing the 8 CH data acquisition system through the SPI communication protocol; and in the fifth step, the universal time coordinate (UTC) time is updated using the universal asynchronous receiver-transmitter (UART) communication protocol and stored in micro-SD memory, and the cycle is repeated.

## 4. Methodology for Propagation Detection of PQDs

**Figure 8:**
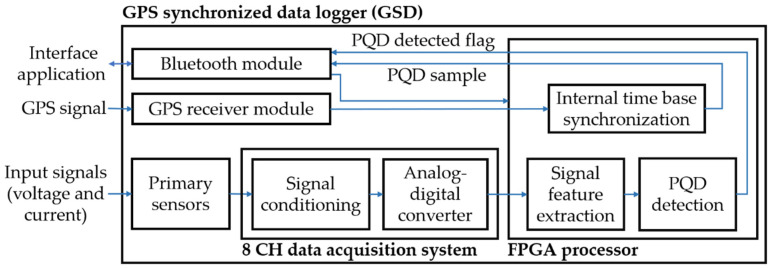
Diagram of disturbance propagation monitoring procedure using GPS-synchronized data loggers called GSD and full parameterized PQD model.

The methodology for the monitoring of PQD propagation is performed by the use of the GPS data logger synchronization procedure reported in Section 3, as well as the full parameterized PQD model reported in [1,7], in which a hybrid method based on GAs and PSO for the estimation of the parameters of Equation (1) is used to automate the detection process. The methodology for PQD propagation detection has been implemented on the FPGA contained in the proprietary GPS synchronized data logger (GSD), as shown in Figure 8; the diagram shows the general blocks that integrate the proprietary PQD detection system. The Bluetooth module is responsible for receiving the instructions from the mobile application, and transmitting the measured signal data for visualization. The GPS receiver module is responsible for providing the PPS synchronized to the data logger. The current and voltage input signals are detected by the primary sensors for conditioning and digitizing in the 8-channel data acquisition system. The digitized current and voltage signals are processed by the signal feature extraction block, which is implemented inside the FPGA processor, to estimate the parameters of Equation (1), and the PQD detection block is responsible for disturbance detection using the parameters extracted from the previous block, obtaining a flag that indicates whether a disturbance has been detected and the sample number of the location of the disturbance in the signal.

**Figure 9:**
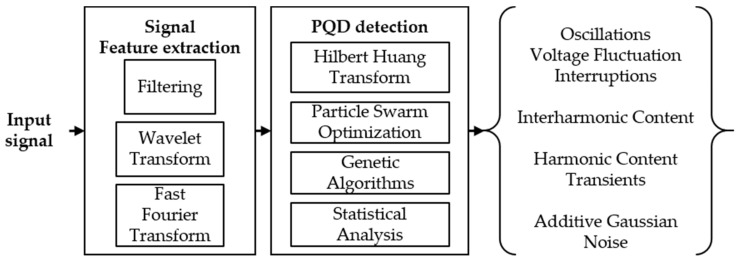
Block diagram of extraction and detection of PQDs.

Figure 9 shows the block diagram of the signal feature extraction and PQD detection sections in general. The diagram has a voltage or current input signal: in the first block, the signal is segmented into different frequencies, using techniques such as low-pass, band-pass, and high-pass filters. Then, the signal provided by the band-pass filter is segmented by the WT, using Equation (2), to separate the transients from the harmonic and interharmonic content, which are separated using the fast Fourier transform (FFT). The second block of the diagram is responsible for classifying the disturbances according to the signals segmented in the previous block, and Equations (3)–(5) of the HHT are used to detect if there are oscillations, voltage fluctuations, and signal interruptions coming from the low-pass filter. GAs are used to classify the harmonic content and transients, PSO is used to classify the interharmonic content, and the statistical analysis allows the classification of the additive Gaussian noise.

**Figure 10:**
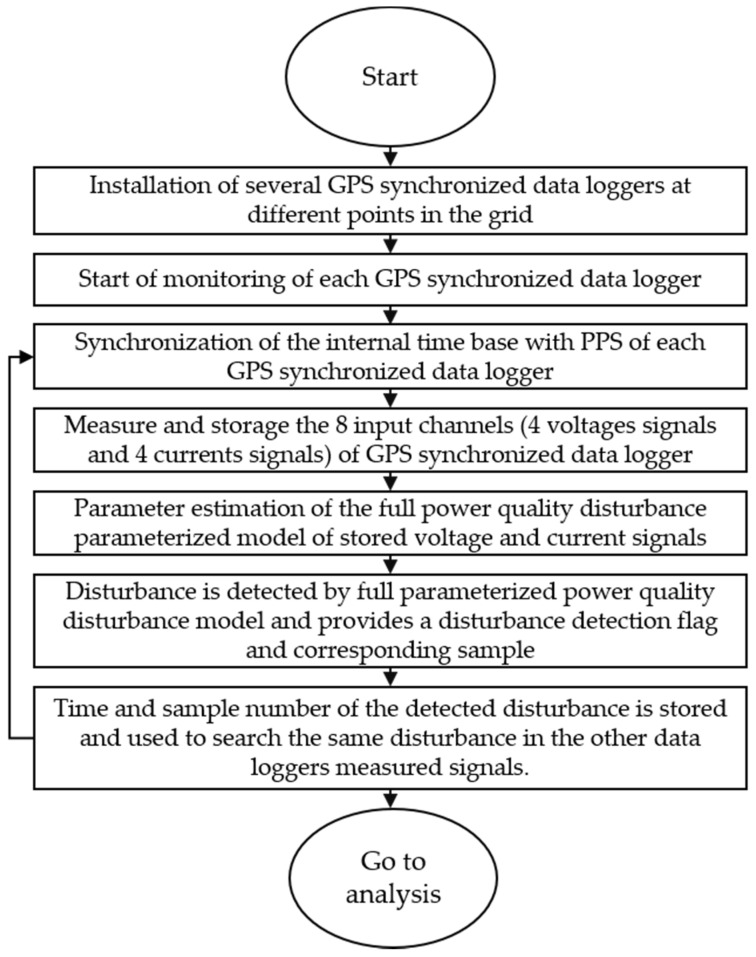
Diagram of the procedure for monitoring a disturbance that propagates in the grid implemented in GSD.

Figure 10 shows the corresponding steps to detect the propagation of PQDs in the grid using the GSD, the first step being the installation of the GPS synchronized data logger in different locations of interest in the grid. The second step consists of the initialization of the monitoring of each GPS synchronized data logger, and continues with the third step that is the synchronization of the internal time base with the PPS of each data logger; at this stage, the signals measured are synchronized. The fourth step is based on the measurement and storage of the eight input channels of the synchronized GPS. The fifth step consists of the analysis of the voltage and current signals using the full PQD parameterized model; in this step, the hybrid approach processes the voltage and current signals, and performs its decomposition using WT, FFT, HHT, GAs, PSO, and statistical analysis, the purpose of which is to analyze it term by term. The model parameters are estimated by minimizing the error between the original signal under analysis and the parameterized signal from the analytical of Equation (1). In the sixth step, the parameters obtained in the previous step are used to determine whether there has been a disturbance, and thus generate a flag indicating the sample number of the disturbance. Finally, in the seventh step, the sample number and time of the disturbance found are used to determine the presence of the same disturbance in the other data loggers, sending information obtained by the GSD to the developed application, which compares the times amongst similar disturbances detected: should these times be within a period of 5 s, it is considered as the propagation of that disturbance, and so, the internal time base is synchronized with the PPS to repeat the cycle again.

## 5. Results

This section shows the result analysis of the proposed methodology by performing the test with different disturbances detected and tracked in the grid using the procedure described in the previous section; the first subsection describes the experimental setup, the second subsection describes the transient analysis, the third subsection describes the voltage fluctuation analysis, and the fourth subsection describes the harmonic content analysis.

### 5.1. Validation Setup

**Figure 11:**
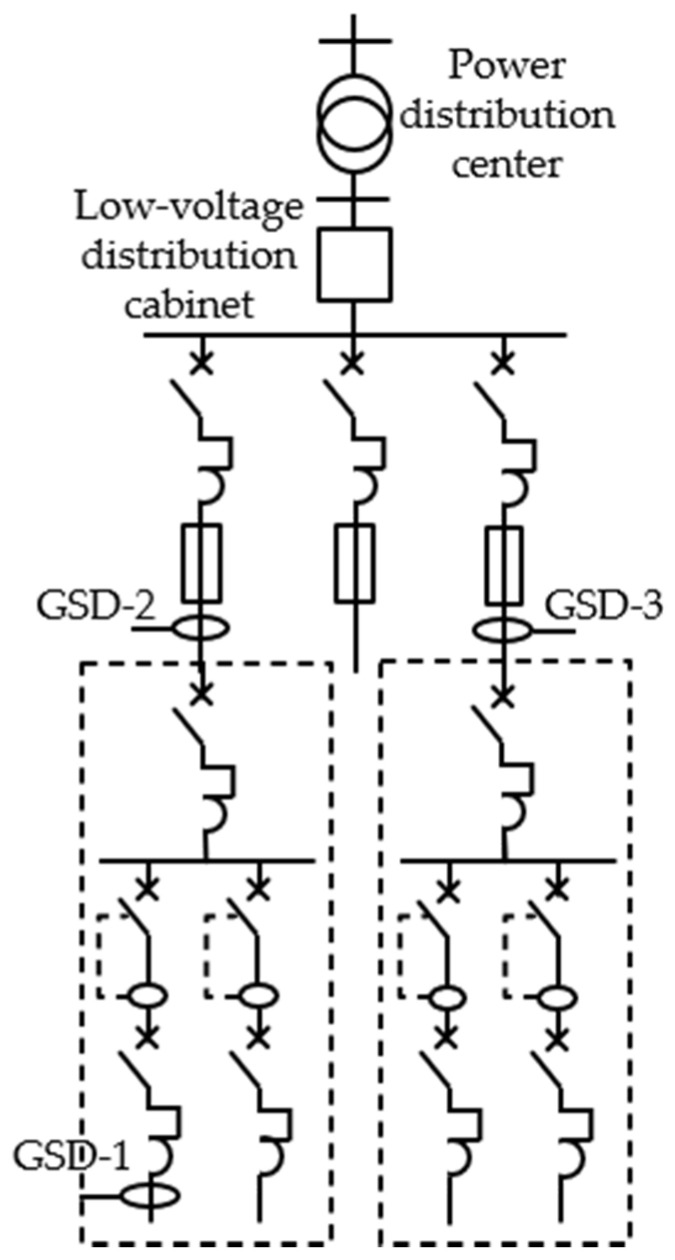
Diagram of the installation of the GPS synchronized data logger (GSD-1, GSD-2, and GSD-3) in different locations of the grid.

For the experimental setup of this work, three proprietary GPS synchronized data loggers, called GSD-1, GSD-2, and GSD-3, were installed at different locations in the industrial facility grid, as shown in the schematic diagram in Figure 11.

### 5.2. Transient Analysis

**Figure 12:**
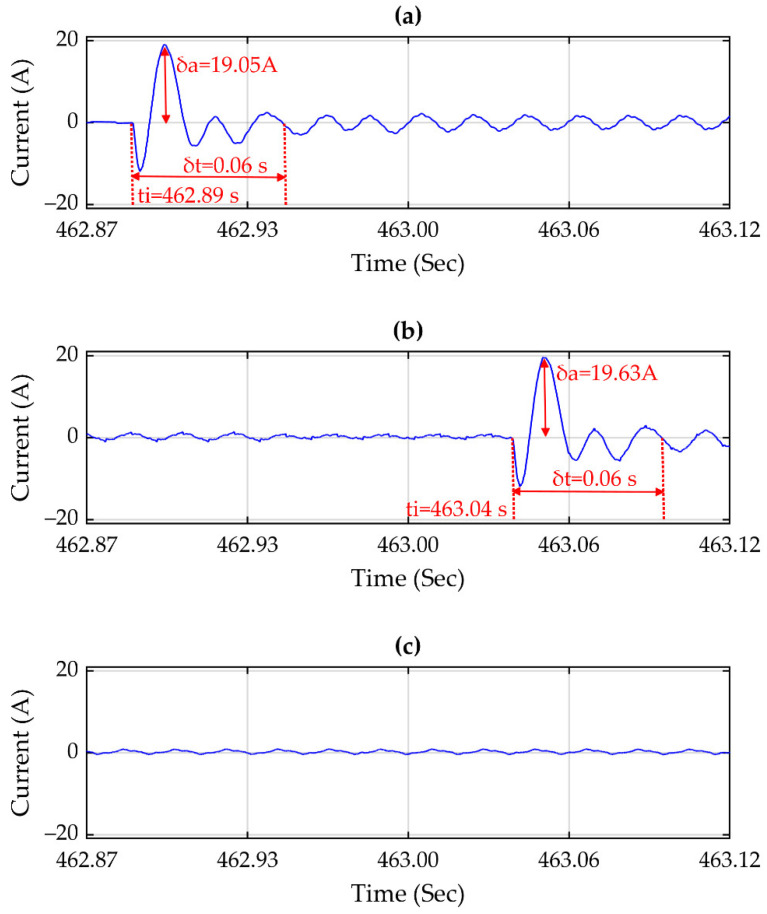
Signals (**a**–**c**) correspond to GPS synchronized data logger GSD-1, GSD-2, and GSD-3, and the graphics show an impulsive transient propagated to two locations on the grid.

Figure 12a–c show the propagation tracking of an impulsive transient disturbance; this disturbance is propagated to two locations in the grid shown in Figure 11. The impulsive transient is caused by an electric motor starting at the location where GSD-1 has been installed. This disturbance is propagated to the location of GSD-2, being the main distribution panel of the line where the electric motor in question is located. Yet, this disturbance has not propagated to the location of the GSD-3 because it is located on another line of the grid.

Figure 12a,b show the characteristics of the propagated disturbance. The impulsive transient observed in Figure 12a has an amplitude (δa) of 19.05 A and a duration (δt) of 0.06 s. On the other hand, the disturbance in Figure 12b has an amplitude (δa) of 19.63 A and a duration (δt) of 0.06 s, which shows that the amplitude of the disturbance in Figure 12b has an increase of 0.58 A compared with the disturbance in Figure 12a. However, the duration times of both transients remain the same. The disturbance in Figure 12b is delayed by 0.15 s after it is caused. The start time (ti) of the disturbance in (a) takes place at 462.89 s, and the beginning time (ti) of the disturbance in (b) is at 463.04 s; with ground on these similar characteristics, it can be determined that it is the same disturbance.

The detection of this disturbance has been performed using the full PQD parameterized model of Equation (1), in which techniques have detected this disturbance using GAs and PSO. The part of Equation (1) that represents this disturbance is shown in Equation (6), corresponding to the transient phenomena, where $\alpha_{m},\;\mathrm{and}\;\beta_{m}$ define the time value when the transient starts and ends, respectively, $c_{m}$ is the amplitude factor, $f_{m}$ is the frequency value, and $\psi_{m}$ is the phase value.

(6) $$x\left(t\right)=\overset{M}{\underset{m=1}{\sum }}c_{m}\left[u\left(t-\alpha_{m}\right)-u\left(t-\beta_{m}\right)\right]*e\left[\left(-\frac{t-\alpha_{m}}{\tau_{m}}\right)\right]*\cos\left[2\pi f_{m}t+\psi_{m}\right]$$

### 5.3. Voltage Fluctuation Analysis

**Figure 13:**
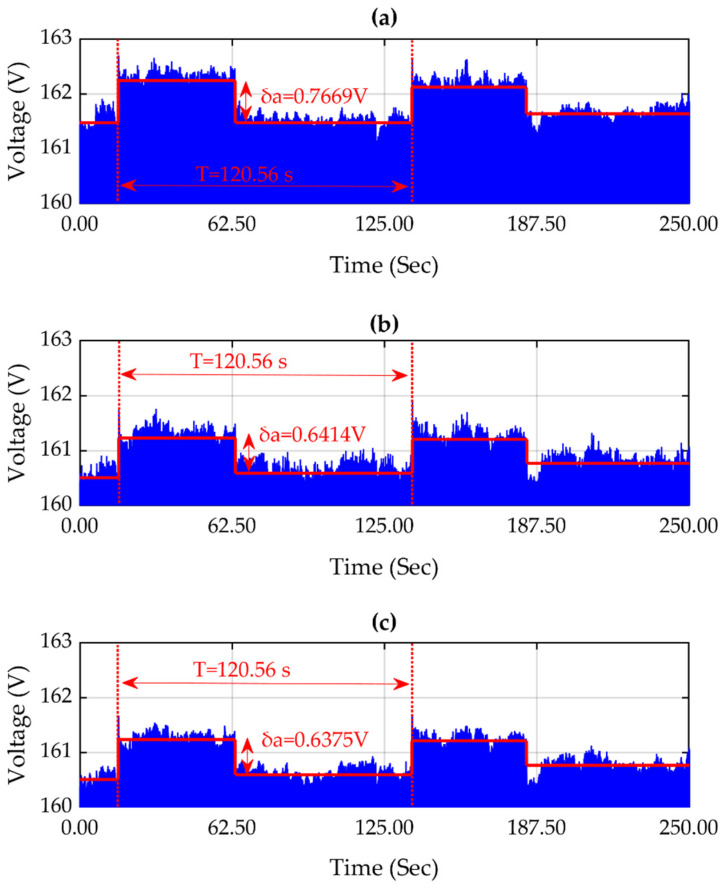
Signals (**a**–**c**) correspond to GPS synchronized data logger GSD-1, GSD-2, and GSD-3, and show a voltage fluctuation disturbance in voltage that propagates to three locations.

Figure 13a–c display the propagation tracking of a voltage fluctuation disturbance; this disturbance is propagated throughout the three locations in the grid shown in Figure 11. Voltage fluctuation is caused by loads with high demand variation, such as inverters, arc furnaces, etc. Accordingly, the voltage fluctuation is reflected in the location of GSD-1, GSD-2, and GSD-3.

For the detection of the voltage fluctuation, the methodology makes use of the part in the full PQD parameterized model, from Equation (1), which corresponds to the phenomena related to the amplitude of the fundamental frequency and its harmonics, represented by Equation (7), where A is the peak value of the amplitude, δ(t) is a time dependent function that stands for events associated with the amplitude disturbances such as oscillations, voltage fluctuation, and interruptions, $a_{h}$ is the time-dependent amplitude factor, $f_{0}$ is the value of the fundamental frequency, $\theta_{1}$ is the value of the phase for the fundamental component, and h is the index value for the h-th harmonic.

(7) $$x_{1}\left(t\right)=A*\left[1+\delta \left(t\right)\right]\left[\cos\left(2\pi f_{0}t+\theta_{1}\right)+\overset{N}{\underset{h=2}{\sum }}a_{h}\left(t\right)\cos\left(2\pi hf_{0}t+\theta_{h}\right)\right]$$

Figure 13a–c show the characteristics of the disturbance; in Figure 13a, the voltage fluctuation has an amplitude (δa) of 0.7669 V and a period (T) of 120.56 s; for Figure 13b, the voltage fluctuation has an amplitude (δa) of 0.6414 V and a period (T) of 120.56 s; and for Figure 13c, the voltage fluctuation also has an amplitude (δa) of 0.6375 V and a period (T) of 120.56 s. As the disturbance characteristics at the three locations are similar, and there is no delay between them, the disturbance is determined to be the same.

### 5.4. Harmonic Content Analysis

**Figure 14:**
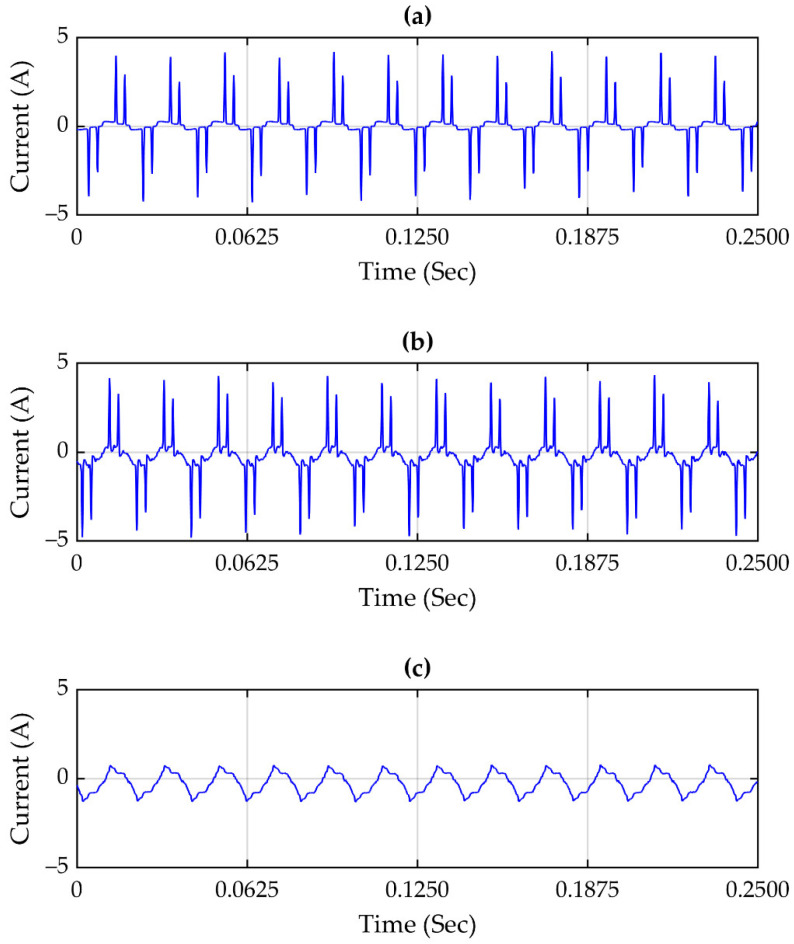
Signals (**a**–**c**) correspond to GPS synchronized data logger GSD-1, GSD-2, and GSD-3, and the graphics show a harmonic content tracking where it propagated to three sites in the grid.

Figure 14a–c show the propagation tracking of the harmonic content; this disturbance is propagated to the three locations in the grid depicted in Figure 11. Harmonic content is caused by non-linear loads which produce a signal distortion. In this regard, harmonic content is propagated to the location of GSD-1, GSD-2, and to a lower proportion to GSD-3. The plots in Figure 14 illustrate the amplitude spectrum corresponding to signals Figure 14a–c, respectively.

Figure 14a–c show the harmonic content in current signals; in Figure 14a,b, the signals are very similar, but Figure 14c has a difference—this is because, according to the location of the data logger, GSD-1 and GSD-2 are on the same line, and GSD-3 is located on a different line. However, through the analysis of the amplitude spectrum of each signal, it is observed that the ones corresponding to 15a–b both have the same frequencies and similar amplitudes. Nevertheless, the amplitude spectrum corresponding to signal 15c has the same frequencies but with a much smaller amplitude; this indicates that the harmonic content propagates to the three locations, but in the GSD-3 location, the propagation occurs in a smaller proportion.

Equation (7) also allows the detection of harmonics as the model represents the phenomena related to the amplitude of the fundamental frequency and harmonics, which is part of the full PQD parameterized model of Equation (1).

## 6. Discussion

The results obtained show the detection of different PQDs that propagated to other locations in the grid. Figure 12 shows a transient that is propagated only to two monitored locations of the grid because the transient is due to the starting of an electric motor, and this disturbance is generated in a point of a branch of the grid and propagated through it. However, it did not propagate to other branches.

**Figure 15:**
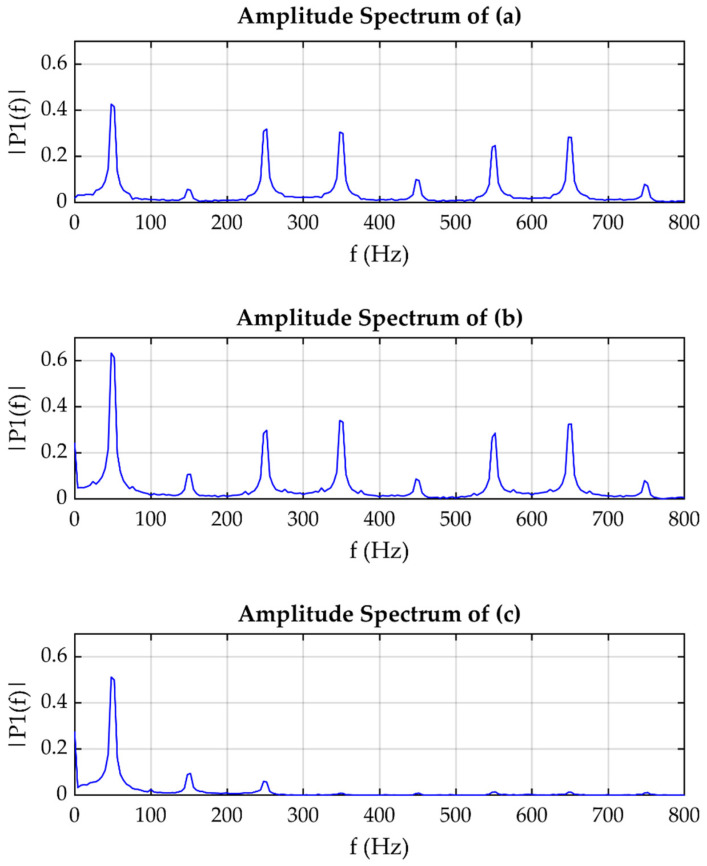
Frequency spectrum plots of the signals corresponding to (**a**–**c**) are shown.

Figure 13 shows a voltage fluctuation disturbance. In this, it can be observed that the disturbance has been propagated to the three monitored points of the grid, which means that this type of disturbance can propagate between branches. This is caused by the switching of nonlinear loads, and can be reflected in the flickering of the lighting. Moreover, Figure 14 and Figure 15 show the propagation of harmonic content through all monitored points of the grid.

The results obtained corroborate that the developed methodology and the synchronization of measurements of electrical signals allow the detection of PQDs and their propagation in a grid; this can be used to track the propagation of disturbances to other points within a grid.

## 7. Conclusions

The propagation of PQDs can cause severe damages to sensitive equipment connected at long distances away from the disturbance’s point of origin. In this work, a system has been developed that allows the detection of the propagation of electrical disturbances using a methodology based on a FPGA sensor that also allows measurements synchronized with GPS.

The development of a proprietary system solves some limitations of other works, shown in Table 2. They include the implementation of models and methodologies that allow an online detection of several PQDs, in addition to methodologies that allow the tracking of the propagation of disturbances with the synchronization of the measurements using GPS.

The proposed system and methodology fully prove their performance by being tested in an industrial installation, where disturbances such as transients, voltage fluctuation, and harmonic content are detected and tracked; regarding transients, they propagated to one site, whereas in the case of voltage fluctuation it propagated to two sites, and in the case of harmonic content it propagated to two sites. For further work, we intend to use this methodology to track PQDs in other areas or facilities where there are other types of loads that generate other types of disturbances.

The integration and use of the PPS and the proprietary data logger allow the synchronization of measurements taken at different sites in the grid, while the implementation of disturbance detection techniques allows the detection of disturbances and the monitoring of whether disturbances propagate to other sites in the grid. However, the system has the limitation of memory for raw data storage to a micro-SD slot, which limits the disturbance monitoring time up to about two weeks.

In further works, we intend to improve the interface of the application that allows us to visualize and control the systems, and implement a second micro-SD memory to extend the time of continuous monitoring. We also plan to perform more tests in industrial facilities to detect other types of disturbances.

## Figures and Tables

**Table 1:** Proprietary data logger hardware technical specifications.

Component	Model	Feature	Functionality
FPGA	Xilinx^®^ FPGA Spartan6 (XC6SLX16)	14579 Logic Elements. 186 Input/Output. Voltage supply: 1.2 V. Integrate Memory: 576 kbit.	It is the main core of the data logger—it coordinates all the modules, controls the ADC acquisition, synchronizes its internal base with the PPS, and processes the measured signals.
ADC	Texas Instruments^®^ (ADS130E08)	16-bit conversion. 8 input channels. 8000 samples per second. Interface Serial Peripheral Interface (SPI). Architecture Delta-Sigma.	The ADC performs the conversion of electrical signals, this ADC contains 8 channels which are used to measure three-phase electrical signals, and its cost–benefit ratio is acceptable for the application.
GPS module receiver	Blox Neo 6M-0-001	Position accuracy: ±2 m. Communication protocol: National Marine Electronics Association (NMEA), Radio Technical Commission for Maritime Services (RTCM). Sensibility del receptor: −161 dBm. Voltage supply: 2.7–3.6 VCC. Provides PPS signal.	This low-cost GPS module is responsible for providing the PPS for the synchronization of measurements, and it is a generic module which is easily accessible to the public.
Bluetooth module	BlueCore 4-External BC417143BGO	Bluetooth 2.4 GHz. Enhanced data rate to 3 Mbps. + 6 dBm Radio frequency transmit power. Standard Human Computer Interaction (HCI).	The Bluetooth module maintains communication with the mobile interface, the cost–benefit ratio is appropriate for the application, and it is easily accessible to the public.

**Table 2:** Performance comparison of the devices used in different works.

System	Capabilities	Disadvantages
PMU [22]	PMU can directly measure frequency, voltage, and current waveforms along with phase angle differences at high sampling rates, and with great accuracy. PMU utilizes a GPS reference source to provide the required synchronization across wide geographical areas.	Does not capture the raw waveform of voltage and current. Does not calculate PQD indices. High cost per unit.
PQ Analyzer [16,19,21]	PQ analyzer calculates some PQ indices in real time. Records electrical signals when it detects a disturbance.	Stores only PQDs detected, does not store the raw data of current and voltage signal. Performs punctual measurements in the grid. High cost per unit.
GSD, our proposal	Continuous measurement of raw waveform of voltage and current. GPS synchronization with PPS obtaining 1 error sample in 24 h during a PPS interruption. PQD detection on-line using GAs and PSO. PQD propagation monitoring. Low cost per unit.	Manual recovery of raw data to post-processing. Data storage limitation to 120 GB, approximately 2 weeks of monitoring.

## Data Availability

No new data were created or analyzed in this study. Data sharing is not applicable to this article.
